# Mosquitocidal Effect of *Glycosmis pentaphylla* Leaf Extracts against Three Mosquito Species (Diptera: Culicidae)

**DOI:** 10.1371/journal.pone.0158088

**Published:** 2016-07-08

**Authors:** Govindaraju Ramkumar, Sengodan Karthi, Ranganathan Muthusamy, Ponnusamy Suganya, Devarajan Natarajan, Eliningaya J. Kweka, Muthugounder S. Shivakumar

**Affiliations:** 1 Molecular Entomology Lab, Department of Biotechnology, Periyar University, Salem- 636 011, Tamil Nadu, India; 2 Natural Drug Research Laboratory, Department of Biotechnology, Periyar University, Salem- 636 011, Tamil Nadu, India; 3 Division of Livestock and Human Diseases Vector Control, Mosquito Section Tropical Pesticides Research Institute, P.O. Box 3024, Arusha, Tanzania; 4 Department of Medical Parasitology and Entomology, Catholic University of Health and Allied Sciences, P.O. Box 1464, Mwanza, Tanzania; Instituto Nacional de Salud Pública, MEXICO

## Abstract

**Background:**

The resistance status of malaria vectors to different classes of insecticides used for public health has raised concern for vector control programmes. Alternative compounds to supplement the existing tools are important to be searched to overcome the existing resistance and persistence of pesticides in vectors and the environment respectively. The mosquitocidal effects of *Glycosmis pentaphylla* using different solvents of acetone, methanol, chloroform and ethyl acetate extracts against three medically important mosquito vectors was conducted.

**Methods:**

*Glycosmis pentaphylla* plant leaves were collected from Kolli Hills, India. The WHO test procedures for larval and adult bioassays were used to evaluate extracts against mosquito vectors, and the chemical composition of extracts identified using GC-MS analysis.

**Results:**

The larvicidal and adulticidal activity of *G*. *pentaphylla* plant extracts clearly impacted the three species of major mosquitoes vectors. Acetone extracts had the highest larvicidal effect against *An*. *stephensi*, *Cx*. *quinquefasciatus* and *Ae*. *aegypti* with the LC_50_ and LC_90_ values of 0.0004, 138.54; 0.2669, 73.7413 and 0.0585, 303.746 mg/ml, respectively. The LC_50_ and LC_90_ adulticide values of *G*. *pentaphylla* leaf extracts in acetone, methanol, chloroform and ethyl acetate, solvents were as follows for *Cx*. *quinquefasciatus*, *An*. *stephensi* and *Ae*. *Aegypti*: 2.957, 5.458, 2.708, and 4.777, 3.449, 6.676 mg/ml respectively. The chemical composition of *G*. *pentaphylla* leaf extract has been found in 20 active compounds.

**Conclusions:**

The plant leaf extracts of *G*. *pentaphylla* bioactive molecules which are effective and can be developed as an eco-friendly approach for larvicides and adulticidal mosquitoes vector control. Detailed identification and characterization of mosquitocidal effect of individual bioactive molecules ingredient may result into biodegradable effective tools for the control of mosquito vectors.

## Background

Control of malaria vectors has entered a new phase of challenge to public health practitioners due to increased threat of insecticides resistance among vectors [1–4]. Large proportions of the human population has been annually affected by vector-borne diseases. Effective vector control includes both larval and adult mosquito control measures, which have great impact in disease control. Synthetic insecticides are currently the most widely used tool for fighting against malaria vectors. However, these synthetic chemicals pose threat against non-target organisms and increase in resistance against targeted vector population [5]. Biopesticides have shown promise of being a source for the environmental eco-friendly and an alternative to synthetic pesticides [6], and for several decades plants have been a source of traditional medicine with least toxicity to mankind and domesticated animals [7,8]. Plant based extracts are presumed to have potential high toxicity and eco-friendly compared to synthetic insecticides for vector of veterinary and medical significance [9]. The current methods used for the control of mosquito-borne diseases -are, the use of insecticides for Indoor residual spray, and long lasting insecticidal nets. It has also provoked undesirable effects, including toxicity to non-target organisms, and fostered environmental and human health concerns [10]. Plant material extracts have been found to be useful and effective as larvicides, repellent or deterrent agents, oviposition attractants or repellent and insect growth hormone regulators [8,11,12]. Plant based materials, whole plants or their extracts have been potentially used for killing and repelling mosquitoes and other nuisance vectors [13]. Previous studies revealed that, different solvents such as acetone, petroleum ether, ethyl acetate and methanol used for extracting active ingredients of *C*. *dentata* had also attributed to effect differences on larvicidal activity against mosquitoes [14,15].

*Glycosmis pentaphylla* (Family: Rutaceae) is an evergreen shrub commonly called an orange berry grown in eastern Asia in Bangladesh, India, and Thailand. *G*. *Pentaphylla* (Rutaceae) is used for treatments of boils, chest pain, hookworm infestation and urinary tract infections in human [16]. Juice extracted from *G*. *pentaphylla* leaves is used for treatment of fever and liver problems. A decoction of roots is used for the facial inflammations treatment [17]. The roots pounded and mixed with sugar are used as an antidote and fever treatment regime [17]. *G*. *Pentaphylla* is reported to contain arborinine among active ingredients [18]. Leaf extract and crude alkaloid possesses antibacterial and antifungal properties [19].

The current study assessed the mosquitocidal activity of different extracts of *Glycosmis pentaphylla* against larvae and adults of three mosquito species.

## Materials and Methods

### Collection of plants

**Fig 1:**
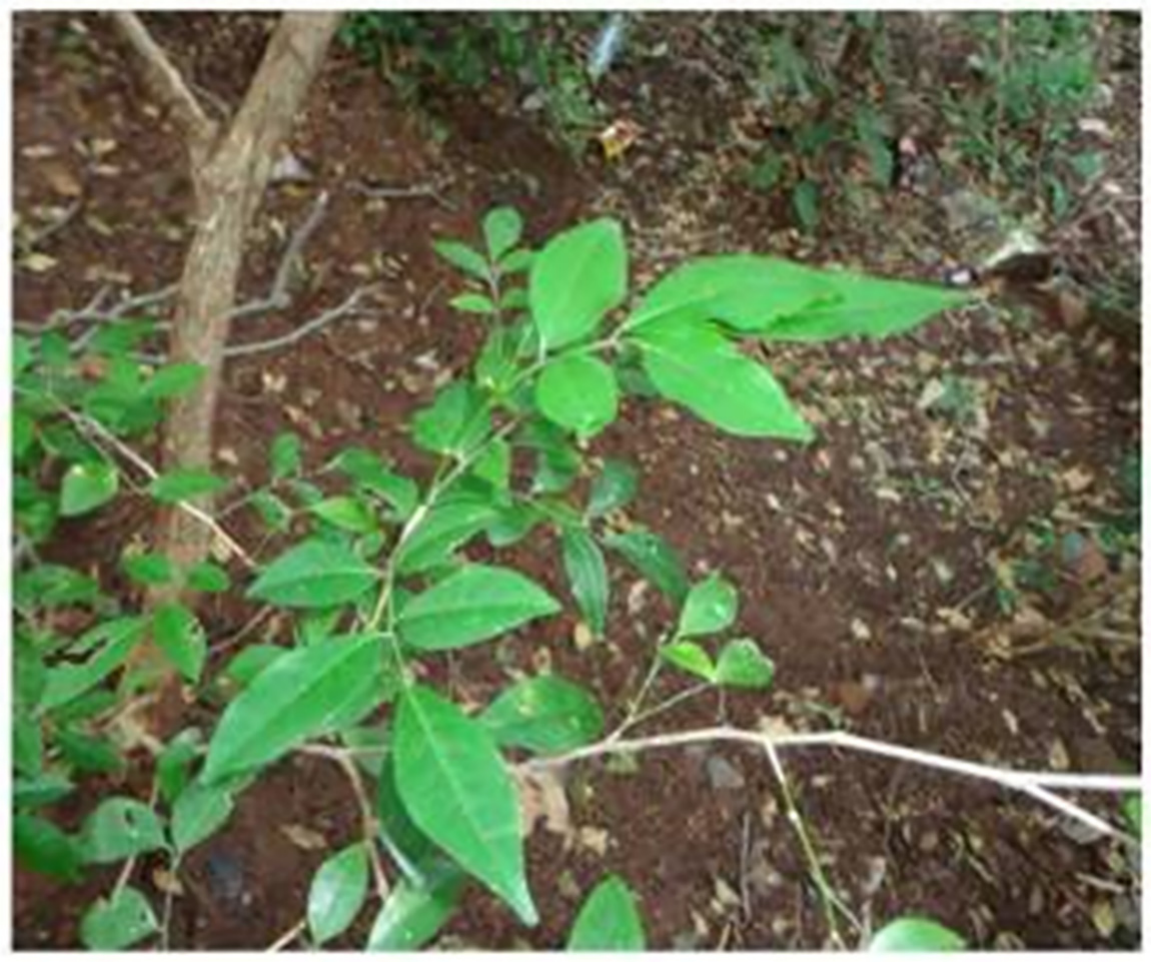
Picture of Glycosmis pentaphylla (Retz) plant picture in the field.

The plant leaves of *Glycosmis pentaphylla* (Fig 1) were sampled from Kolli Hills of Eastern Ghats in a region that is rich in biodiversity and indigenous populations. Kolli Hills are located in the Namakkal district which lies between 10°12'–11°7'N, and 76°–77°56'E with an altitude 1300 meters above sea level and the taxonomic identification of plant was made by Dr. D. Natarajan, Assistant Professor, from Department of Biotechnology, Periyar University, Tamil Nadu, India, did the taxonomic identification of the plant and the voucher specimen was deposited in the herbarium. Periyar university approved ethical clearance for this research. In Kolli Hills, the management was informed about the study objectives and written consent was obtained with permission to collect plant material. This study did not involve any endangered or highly protected species.

### Plant extracts preparations

Plant leaves were dried under shade for a period of 7 to 10 days in the shade at environmental temperatures (27–37°C daytime). A blander was used for powdering the dried leaves of which three hundred grams were extracted separately using the following chemicals for eight hours, chloroform, ethyl acetate, acetone and methanol in a Soxhlet apparatus with boiling point ranging between 50–80°C. The leaf extracts were concentrated at a pressure of 22–26mm Hg at 45°C. The obtained active ingredients were stored in a room temperature (in a dark room).

### Mosquitoes culture

The National Centre for Disease Control (NCDC), Mettupalayam, Tamil Nadu, India provided larvae of *Anopheles stephensi*, *Aedes aegypti and Culex quinquefasciatus* for this study. The larvae were kept in plastic trays containing tap water was used for rearing under laboratory conditions at 27±2°C and relative humidity of 75–85%. The photophase was maintained at 14:10 light and dark. Ground dog biscuit and yeast powder was used as larvae food in the ratio of 3:1.

### Larvicidal bioassay

WHO standard protocol for evaluating the larvicides was used to evaluate the plant extracts toxicity against mosquito larvae[20], with some minor modifications done by previous studies, [21]. Twenty five (25) fourth instar larvae were transferred to a small disposable paper cups with a mixture of 249 ml of water and 1.0 ml of proposed plant extract. The control used as DMSO. Dosage evaluation started with the lowest dosage of 0.01mg/ml to 2 mg/ml. Larvae mortality was monitored after 24 hours of exposure in all replicates of each dosage. The percentage mortality was reported from three replicates. The LC_50_ (lethal concentration that kills 50% of the exposed larvae) and LC_90_ (lethal concentration that kills 90% of the exposed larvae) values were calculated after 24 h using probit analysis method [22].

### Adulticidal bioassay

Adulticidal bioassay was performed by using a clean glass test tube and different concentrations of five solvent extracts were made ranging from 0.1, 0.3, 0.5, 1 and 2mg/ml was coated with test tube and allow to dry the solvent extract [22–25]. There were three replicates per concentration and twenty unfed female mosquitoes were released in each replicates. Tubes were tightly covered with netting materials and coated with solvent alone served as control. Mosquito mortality was determined 24 hours post exposure. LD_50_, LD_90_ with their 95% confidence limits were determined using Log Probit analysis test [22].

### GC–MS analysis

The plant extracts of *G*. *pentaphylla* were analysed using gas chromatography (Polaris Q Ion Trap GC/FID) and mass spectrometry (Perkin Elmer Q-700 equipment). We followed the method used by previous published research findings [26,27], which used one type of column (preferably Polaris Q Ion Trap GC/FID). The column temperature programme was 35°C for 2 min, increased to 180°C at 4°C/min, then increased to 280°C at 20°C/min. Helium was used as a carrier gas at a 0.9 ml/min. The best mass spectrum was obtained at 70 eV ionization voltage in the machine. Individual compounds were identified using Wiley/ NBS Registry of mass spectral database, the NIST machine (version 3.0) database. The Retention Time (RT) and Kovats Index (KI) values of several authentic reference compounds were compared with isolated compounds for identification.

### Statistical analysis

The mean mortality data were analysed using probit analysis for calculating LC_50_, LC_90_ and other statistics at 95% confidence interval limits of upper and lower confidence limits. The Chi-square values were calculated using the SPSS Statistical software package version 16.0 for windows.

## Results

### Larval and adult bioassay

The larvicidal activity results were obtained from bioassays of the crude extracts by acetone, methanol, chloroform and ethyl acetate solvent leaf extracts of *Glycosmis pentaphylla* against the larvae of three important vector mosquitoes in different concentration varied among species; *Cx*. *Quinquefasciatus* (Table 1), *An*. *stephensi* (Table 2) and *Ae*. *aegypti* (Table 3). Among the plant extracts tested, the highest larvicidal activity was observed in acetone extracted compounds against *Cx*. *quinquefasciatus*, *An*. *stephensi* and *Ae*. *aegypti* with the LC_50_ and LC_90_ values of 0.0005, 138.54; 0.267, 73.741 and 0.058, 303.746 mg/ml, respectively. The 95% confidence limits LC_50_ (95%CI) and LC_90_ (95%CI), chi-square and degree of freedom (df) values were also calculated. In control assays we did not find any significant mortality.

**Table 1:** Larvicidal activity of *Glycosmis pentaphylla* leaf extracts against fourth instar larvae of *Cx*. *quinquefasciatus*, *An*. *stephensi* and *Ae*. *Aegypti*.

Mosquito Species	Extract	n^a^	LC_50_ (mg/ml)	LC_90_ (mg/ml)	χ^2^	df
			(95% CL)	(95% CL)		
*Cx*. *quinquefasciatus*	Acetone	375	0.00045	138.54	5.625*	3
			(3.8E-08-5.367)	(0.061–313)		
*Cx*. *quinquefasciatus*	Methanol	375	0.095	5612.981	6.471*	3
			(0.012–0.752)	(0.235–134147950.9)		
*Cx*. *quinquefasciatus*	Chloroform	375	0.267	13.878	19.374*	3
			(0.039–1.805)	(0.043–4474.45)		
*Cx*. *quinquefasciatus*	Ethyl acetate	375	0.0508	2.458	10.785*	3
			(1.076)	36.182		
*An*. *stephensi*	Acetone	375	0.267	73.741	1.938	3
			(0.469)	(39900.31)		
*An*. *stephensi*	Methanol	375	0.480	34.648	3.147	3
			(0.296–0.767)	(9.258–859.468)		
*An*. *stephensi*	Chloroform	375	0.456	30.801	5.2597*	3
			(0.280–0.717)	(8.6228–648.6936)		
*An*. *stephensi*	Ethyl acetate	375	0.396	14.314	1.679	3
			(0.257–0.572)	(5.529–102.974)		
*Ae*. *aegypti*	Acetone	375	0.0585	303.746	12.328*	3
			(8.7E-05-39.279)	(3.9E-06-2.4E+10)		
*Ae*. *aegypti*	Methanol	375	0.121	6376.845	3.602	3
			(0.019–0.757)	(0.26506)		
*Ae*. *aegypti*	Chloroform	375	0.112	31.385	0.993	3
			(0.014–0.226)	(6.444–7596.35)		
*Ae*. *aegypti*	Ethyl acetate	375	0.204	22.687	3.641	3
			(0.338)	(744.033)		

n^a^—total number of mosquitoes larvae used; n = 25/replicate and three replicate per solvent were taken and five different concentration were used, LC_50_ -Lethal concentration 50% mortality, LC_90_- Lethal concentration 90% mortality, LCL- lower confidence limits, UCL- upper confidence limits, χ2-chi square, df—degrees of freedom (**Note:** Chi-square values with astaric are significant P>0.05).

**Table 2:** Adulticidal activity of plant leaf extract of *Glycosmis pentaphylla* against *Cx*. *quinquefasciatus*, *An*. *stephensi* and *Ae*. *Aegypti*.

Mosquito Species	Sample	n^a^	LC_50_ (mg/ml)	LC_90_(mg/ml)	χ^2^	df
			(95% CL)	(95% CL)		
*Cx*. *quinquefasciatus*	Acetone	300	4.518	8.439	3.040	3
			(2.859–19.250)	(5.063–39.142)		
*Cx*. *quinquefasciatus*	Ethyl Acetate	300	4.217	7.483	2.382	3
			(2.821–11.938)	(4.768–22.889)		
*Cx*. *quinquefasciatus*	Methanol	300	3.572	7.218	4.374*	3
			(2.422–9.406)	(4.625–20.879)		
*Cx*. *quinquefasciatus*	Chloroform	300	2.957	5.458	2.907	3
			(2.244–4.904)	(3.960–9.744)		
*An*. *stephensi*	Acetone	300	4.464	8.752	3.154	3
			(2.776–23.450)	(5.135–50.401)		
*An*. *stephensi*	Ethyl Acetate	300	5.893	9.693	1.437	3
			(3.981–9.421)	(6.0087–14.5431)		
*An*. *stephensi*	Methanol	300	5.139	9.210	0.696	3
			(3.108–38.350)	(5.292–74.515)		
*An*. *stephensi*	Chloroform	300	2.708	4.777	2.641	3
			(2.152–3.961)	(3.649–7.458)		
*Ae*. *aegypti*	Acetone	300	5.139	9.210	0.696	3
			(3.108–38.350)	(5.292–74.515)		
*Ae*. *aegypti*	Ethyl Acetate	300	6.236	11.051	1.209	3
			(4.1167–7.9812)	(9.6110–13.7613)		
*Ae*. *aegypti*	Methanol	300	4.691	8.802	1.351	3
			(2.910–24.590)	(5.174–50.276)		
*Ae*. *aegypti*	Chloroform	300	3.449	6.676	3.213	3
			(2.421–7.620)	(4.449–16.064)		

n^a^—means Number of adults used; n = 20/replicate and three replicates per solvent were taken and five different concentration were used, LC_50_ -Lethal concentration 50% mortality, LC_90_-Lethal concentration 90% mortality, LCL- lower confidence limits, UCL- upper confidence limits, χ^2^-chi square, df—degrees of freedom (**Note:** Chi-square values with astaric are significant P>0.05).

**Table 3:** Chemical composition of Acetone leaf extract from *Glycosmis pentaphylla*.

S. No	RT	Area	Area%	Compound name	Activity
1	28.974	269,734,080.0	36.061	4h-1-Benzopyran-4-One, 2-(3,4-Dimethoxyphenyl)-3,5,6,7-Tetramethoxy	Unknown
2	23.972	127,418,296.0	17.035	Cyclopropanecarboxylic Acid, 2-Methyl-, 2,6-Di-T-Butyl-4-Methylphenyl Ester	insecticidal activity
3	28.799	71,275,064.0	9.529	Unknown	Unknown
4	22.982	47,226,244.0	6.314	2h-1-Benzopyran-2-One, 6-(1-Hydroxy-3-Methylbutyl)-7-Methoxy	Antibacterial activity and anticancer activity
5	19.550	35,565,652.0	4.755	4,4'-ethylenebis(2,6-di-tert-butylphenol)	antihyperlipidemic
6	22.221	35,264,760.0	4.715	4,4'-ethylenebis(2,6-di-tert-butylphenol)	antihyperlipidemic
7	22.967	11,321,852.0	4.650	Hexane,3-(1,5-dimethyl-4-heanyl)-6-metheline-,(s-(R,S)	Pharmacological and antioxidant activity
8	12.577	30,418,032.0	4.067	Benzene, 1-(1,5-dimethyl-4-hexenyl)-4-methyla1	Antioxidant activity
9	14.443	15,103,241.0	2.019	Cis-. Alpha.-copaene-8-OL	Antibacterial activity
10	20.581	14,524,484.0	1.942	Diisopropyl (1-naphthyloxy) silane	Antimicrobial activity

The adulticidal activity of acetone, methanol, chloroform and ethyl acetate solvent leaf extracts of *G pentaphylla* against the adults of three important vector mosquitoes are reported for *Cx*. *quinquefasciatus*, *An*. *stephensi*, and *Ae*. *Aegypti* (Table 2). The plant crude extracts mortality increased proportionally with dosage. Among the tested plant extract, the highest adulticidal effect was found to be in chloroform extract against *Cx*. *quinquefasciatus*, *An*. *stephensi* and *Ae*. *aegypti*. The control was tested without solvent showing no mortality. The LC_50_ and LC_90_ values of *G pentaphylla* leaf extracts of acetone, methanol, chloroform and ethyl acetate against adulticidal activity of *Cx*. *quinquefasciatus*, *An*. *stephensi* and *Ae*. *aegypti* were as follows: 2.957and 5.458;2.708 and 4.777; 3.449 and 6.676 mg/ml, respectively.

### GC-MS analysis and identification of compounds

**Fig 2:**
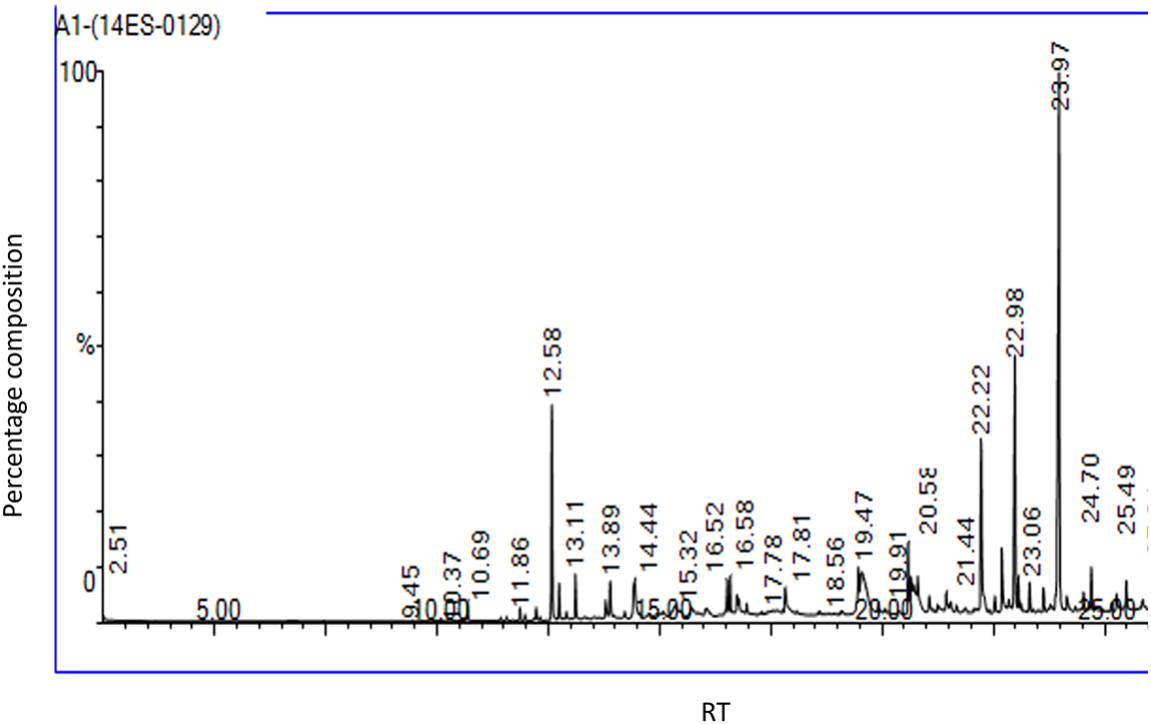
Chromatogram from GC-MS analysis of acetone extracts of *Glycosmis pentaphylla* (ACQUISITION PARAMETERS; Oven: Initial temp 60°C for 2min, ramp 10°C/minto 300°C, hold 6min, Inj Aauto = 250°C, Volume = 0μL,Split = 10:1,Carrier Gas = He, Solvent Delay = 2.00min, Transfer Temp = 240°C, Source Temp = 240°C,Scan:50to 600Da,Column 30.0mx250μm).

**Fig 3:**
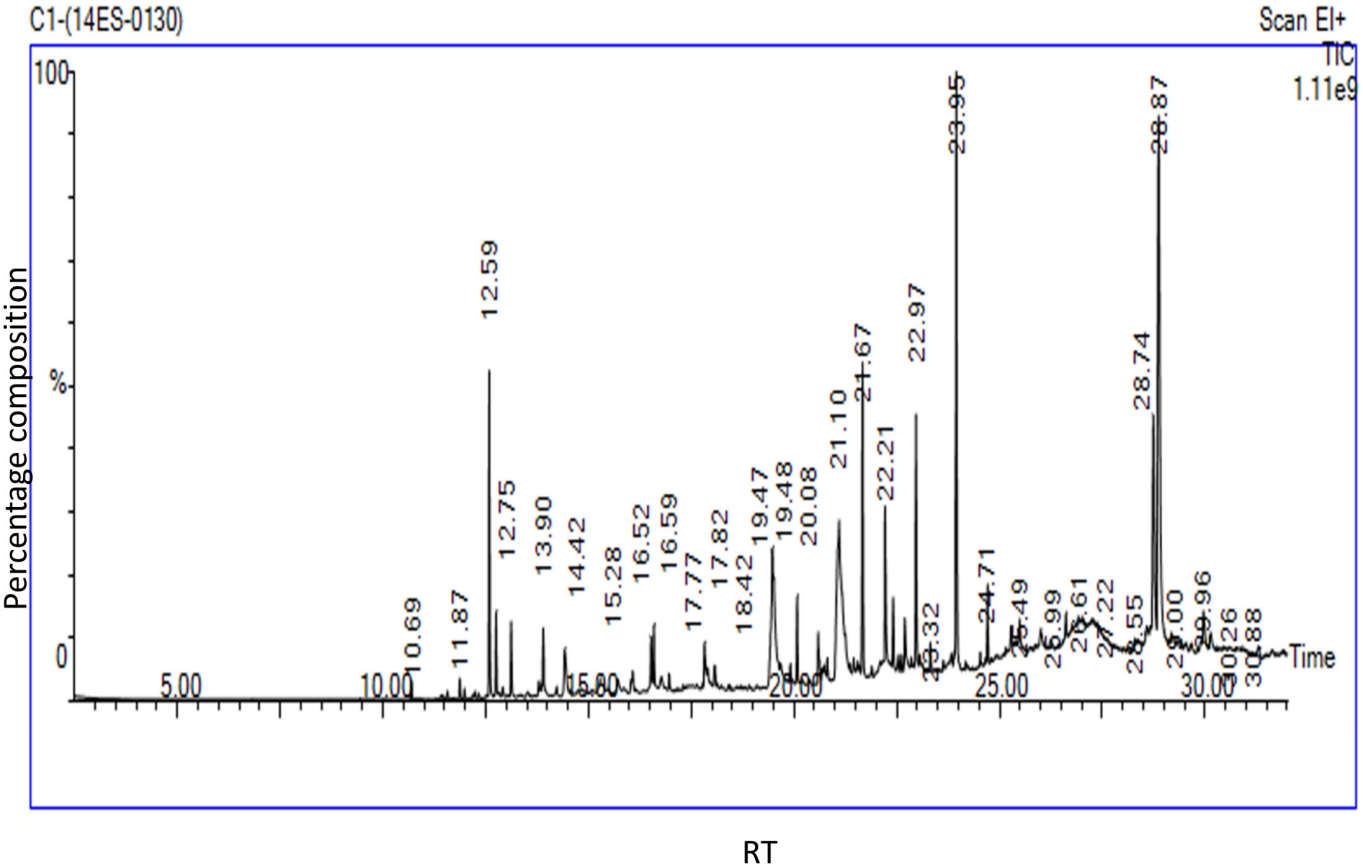
Chromatogram from of GC-MS analysis of chloroform extracts of *Glycosmis pentaphylla* (ACQUISITION PARAMETERS; Oven:Initial temp 60°C for 2min, ramp 10°C/min to 300°C, hold 6min,Inj Aauto = 250°C,Volume = 0μL,Split = 10:1,Carrier Gas = He, Solvent Delay = 2.00min, Transfer Temp = 240°C, Source Temp = 240°C,Scan:50to 600Da, Column 30.0mx250μm).

GC-MS analysis of acetone and chloroform solvent extracts of *G*. *pentaphylla* extracts showed 40 peaks which indicating the presence of 20 phytochemical compounds (Figs 2 and 3). On the comparison of the mass spectra of the constituents with the NIST library, the 20 compounds were characterized and identified (Tables 3 and 4). The major chemical compounds which were identified in acetone solvent extracts based on the 5 highest peaks were 4h-1-Benzopyran-4-one 2-(3,4-Dimethoxyphenyl)-3,5,6,7-Tetramethoxy (36.061%), Cyclopropane carboxylic Acid, 2-Methyl-, 2,6-Di-T-Butyl-4-Methylphenyl Ester (17.035%), 2h-1-Benzopyran-2-One, 6-(1-Hydroxy-3-Methylbutyl)-7-Methoxy(6.314%),4,4'-ethylenebis(2,6-di-tert-butylphenol) (4.775%), Hexane,3-(1,5-dimethyl-4-heanyl)-6-metheline-,(s-(R,S) (4.650%). For the chloroform solvent extracts based on the 5 highest peaks, the identified compounds were 4h-1-Benzopyran-4-One, 2-(3,4-Dimethoxyphenyl)-3,5,6,7-Tetramethoxy (20.326%), Glaucyl Alcohol (15.876%), Cyclopropanecarboxylic Acid, 2-Methyl-, 2,6-Di-T-Butyl-4-Methylphenyl Ester (11.695%), 3-methyl-2-(2-oxopropyl)furan (9.511%), 2r-acetoxymethyl-1,3,3-trimethyl-4t-(3-methyl-2-buten-1-yl)-1t-cyclohexanol (5.923%).

**Table 4:** Chemical composition of chloroform leaf extract from *Glycosmis pentaphylla*.

S/N	RT	Area	Area%	Compound name	Activity
1	28.869	49,491,564.0	20.326	4h-1-Benzopyran-4-One, 2-(3,4-Dimethoxyphenyl)-3,5,6,7-Tetramethoxy	Unknown
2	21.096	38,656,132.0	15.876	Glaucyl Alcohol	Antibacterial, antioxidant activity
3	23.947	28,475,728.0	11.695	Cyclopropanecarboxylic Acid, 2-Methyl-, 2,6-Di-T-Butyl-4-Methylphenyl Ester	insecticidal activity
4	19.475	23,157,604.0	9.511	3-methyl-2-(2-oxopropyl)furan	Antifungal and phytochemical activity
5	21.666	14,421,752.0	5.923	2r-acetoxymethyl-1,3,3-trimethyl-4t-(3-methyl-2-buten-1-yl)-1t-cyclohexanol	insecticidal activity
6	12.587	12,575,728.0	5.165	Benzene, 1-(1,5-dimethyl-4-hexenyl)-4-methyl	Antidermatophytic activity
7	22.211	7,736,138.0	3.177	5-(2,5-dimethoxy-phenyl)-2h-pyrazol-3-ol	Antioxidant and antimicrobial activity
8	14.418	5,192,260.5	2.132	Cyclohexene, 1,5,5-Trimethyl-6-(2-Propenylidene)-	Antimicrobial activity
9	22.691	3,786,916.0	1.555	1,1'-Biphenyl, 2-Formyl-4',5',6'-Trimethoxy	-
10	20.075	3,677,789.0	1.510	Z,Z-6,28-Heptatriactontadien-2-One	Vasodilator, carcinogenic and Antioxidant activity

## Discussion

The findings of this study have shown that phytochemical compounds extracted from *G*. *pentaphylla* might be innovative, alternative to synthetic insecticides in the future as these are safe for non-target organisms. Chemical compounds extracted from different parts of plant have different active ingredients with different activity against larvae and adult mosquitoes. Plant crude extracts might be more effective than individual active compound due to active ingredients synergisms which can be effective in managing resistant population of mosquitoes. Our results show that the crude acetone, methanol, chloroform and ethyl acetate solvent leaf extracts of *G pentaphylla* were effective against the larvae of three important vector mosquitoes, *Cx*. *quinquefasciatus*, *An*. *stephensi* and *Ae*. *aegypti*. These results are similar to previous reported study findings [14,15] who observed the larvicidal activity of *C*. *dentata* plant extract against vector mosquitoes, namely, *Ae*. *aegypti* (LC_50_ = 0.169 mg/ml) *Cx*. *quinquefasciatus* (LC_50_ = 0.150 mg/ml) and *An*. *stephensi* (LC_50_ = 0.046 mg/ml). Previous studies have shown that, petroleum ether of *R*. *nasutus* have stronger larvicidal effect with LC_50_ values ranging from 3.9 to11.5 mg/l, while *Derris elliptica* had LC_50_ values varying from 11.2 to18.84 mg/l against *Ae*. *aegypti*, *Cx*. *quinquefasciatus*, *An*. *dirus*, and *Mansonia uniformis* [28]. Khanna and others found that, crude leaf extracts of *Gymnema sylvestre* had the highest mortalities at the concentration of 1000 ppm against the larvae of *An*. *subpictus* (LC_50_ = 166.28 ppm) and *Cx*. *quinquefasciatus* (LC_50_ = 186.55 ppm). Among its active ingredients, gymnemagenol compound isolated from petroleum ether had LC_50_ of 22.99 ppm for larvae of *An*. *subpictus* and 15.92 ppm for *Cx*. *quinquefasciatus* [29].

In other studies, the limonoids extracted from neem seeds evaluated for larvicidal, pupicidal, adulticidal, and anti-ovipositional activity against *An*. *stephensi* using different solvent had larval mortality which was dose dependent[30]. Ethanol extract induced mortality to *C*. *sinensis* at a concentration of 272.19 ppm (LC_50_) and 457.14 ppm (LC_90_) while for *An*. *stephensi* LC_50_ was 289.62 and LC_90_ was 494.88 ppm. In *Ae*. *aegypti* LC_50_ was 320.38ppm and LC_90_ was 524.57 ppm [30,31]. In the present study, the adulticidal results had the LC_50_ and LC_90_ values were 205.78 and 459.51 ppm for *An*. *stephensi*, 242.52 ppm and 523.73 ppm for *Ae*. *aegypti* respectively which was similar to study by Murugan and others with the extracts from *Citrus* peals [31].

The adulticidal activity results of acetone, methanol, chloroform and ethyl acetate solvent leaf extracts of *G*. *pentaphylla* against adult vector mosquitoes, *Cx*. *quinquefasciatus*, *An*. *stephensi* and *Ae*. *aegypti* showed to have dose-dependent mortality. At higher concentrations, the adult showed restless movement for some times with abnormal wagging and then died. Among the extracts tested, the highest adulticidal activity was observed in chloroform extract against *Cx*. *quinquefasciatus*, *An*. *stephensi* and *Ae*. *aegypti*. The lethal dose to kill 50% and 90% (LC_50_ and LC_90)_ values of *G*. *pentaphylla* leaf extracts against adulticidal activity of (acetone, methanol, chloroform and ethyl acetate), *Cx*. *quinquefasciatus*, *An*. *stephensi* and *Ae*. *aegypti* were 2.957and 5.458; 2.708 and 4.777;3.449 and 6.676 mg/ml. Rajkumar and others found that, the leaf extract of *C*. *asiatica* has larvicidal and an inhibitory properties for the emergence adults against *Cx*. *quinquefasciatus* [32]. The reported results in this study in adult emergence inhibitory and mosquitocidal effects are by far greater that those reported by other studies using different plant extracts. The above findings support the results observed in this study that, plant *G*. *pentaphylla* act as potent larvicide and adulticide as well as disrupting the growth of larvae of *Cx*. *quinquefasciatus*, *An*. *stephensi* and *Ae*. *aegypti*.

The isolation of compounds from leaf extracts of *G pentaphylla*, as described here, could lead to the development of natural mosquitocidal products to replace the synthetic insecticides. The use of natural plant based products by individuals and communities can enhance their better diseases vector control, plant species protection and propagations hence become source of income to community to eradicate poverty[33]. For example, our previous study provides the active ingredients of *C*. *dentata* plant extracts of acetone solvent against mosquito vectors [15]. Our study shows that besides mosquito larval and adult control efficacy, the compounds in the extracts which may be useful as further studies are needed to identify structure of the bioactive molecules.

## Conclusion

In conclusion, the findings of this study have shown a great effectiveness of *Glycosmis pentaphylla* extracts by different solvents against the three major mosquito species in aquatic stages and adults. Our findings showed that leaf extract of *G pentaphylla* bioactive molecules to be an effective and can be developed as an eco-friendly larvicides and adulticides for mosquito vector control. This study suggests that future research work on the use of individual active ingredient to evaluate its mosquitocidal effect on the three species used in this study in semifield and small scale field trials for invention of environmentally safe botanical insecticide for the control of mosquito vectors.
